# Supplementary material for: Designing and Evaluating a Portable UV-LED Vane Trap to Expedite Arthropod Biodiversity Discovery
Source: Insects. 2024 Jan 1;15(1):21. doi: 10.3390/insects15010021 (PMC10816512; doi:10.3390/insects15010021)
Supplement: Supplementary file 1 [file insects-15-00021-s001.zip › Figures S1 and S2.pdf]

Figure S1. Conventional active light trapping equipment

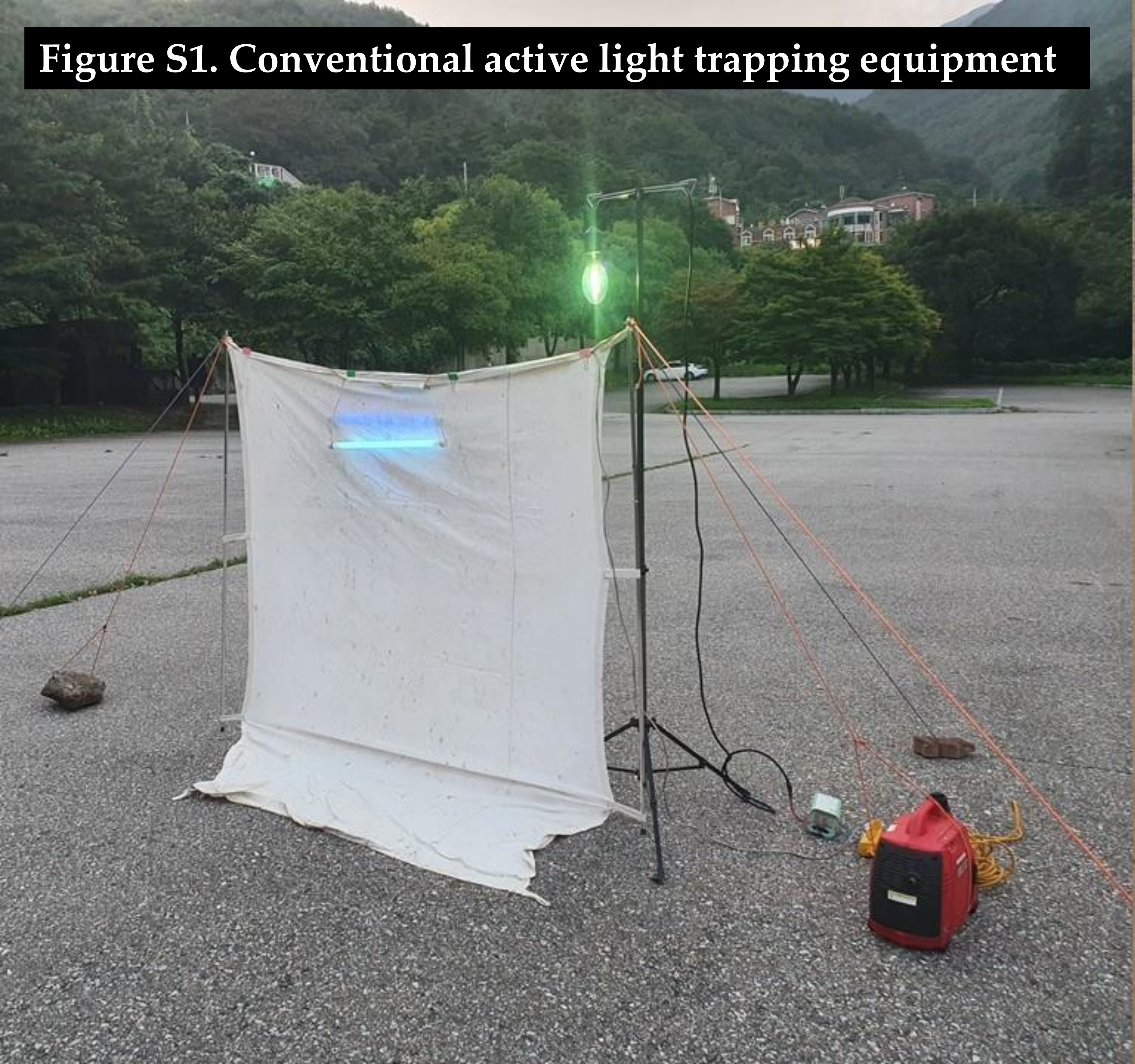

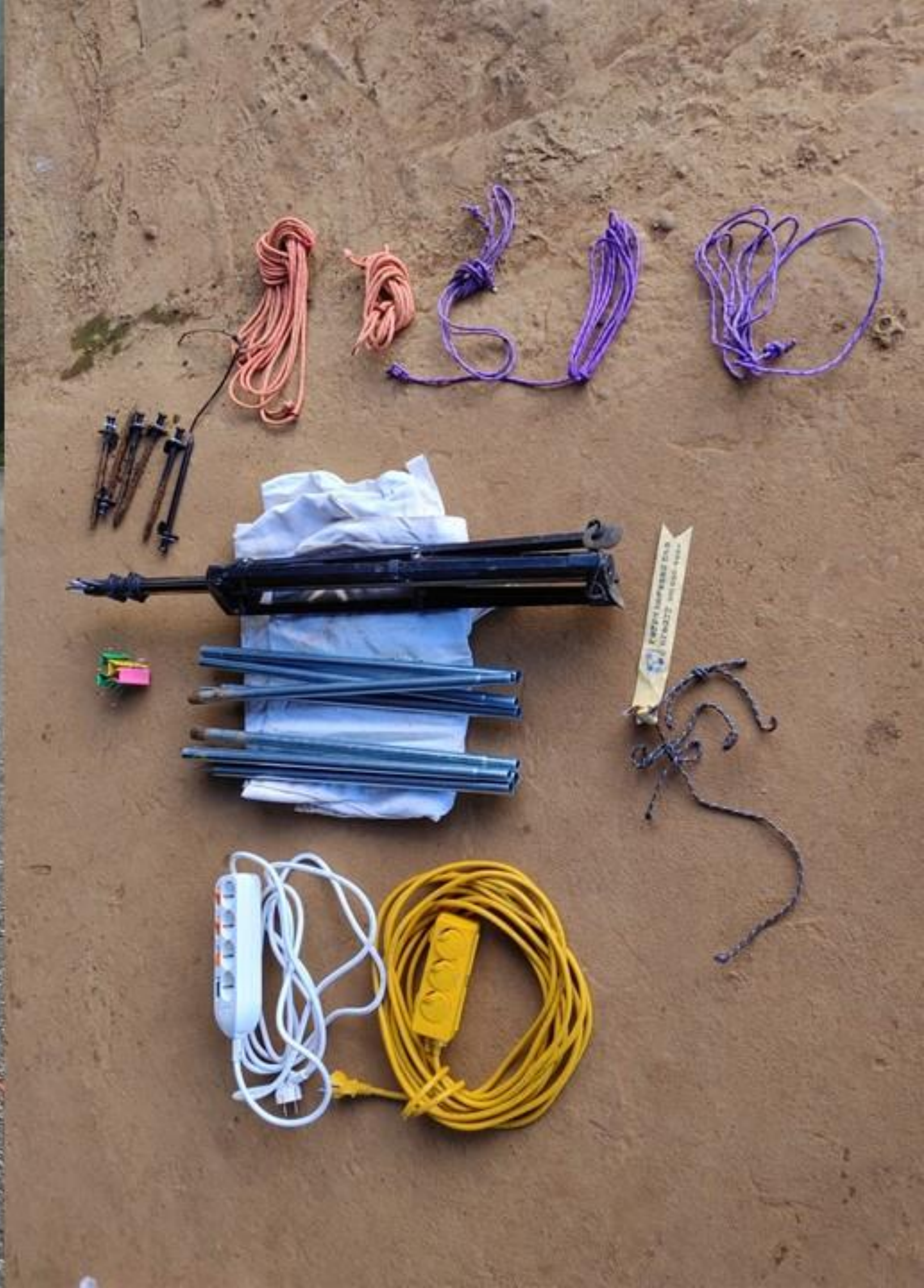

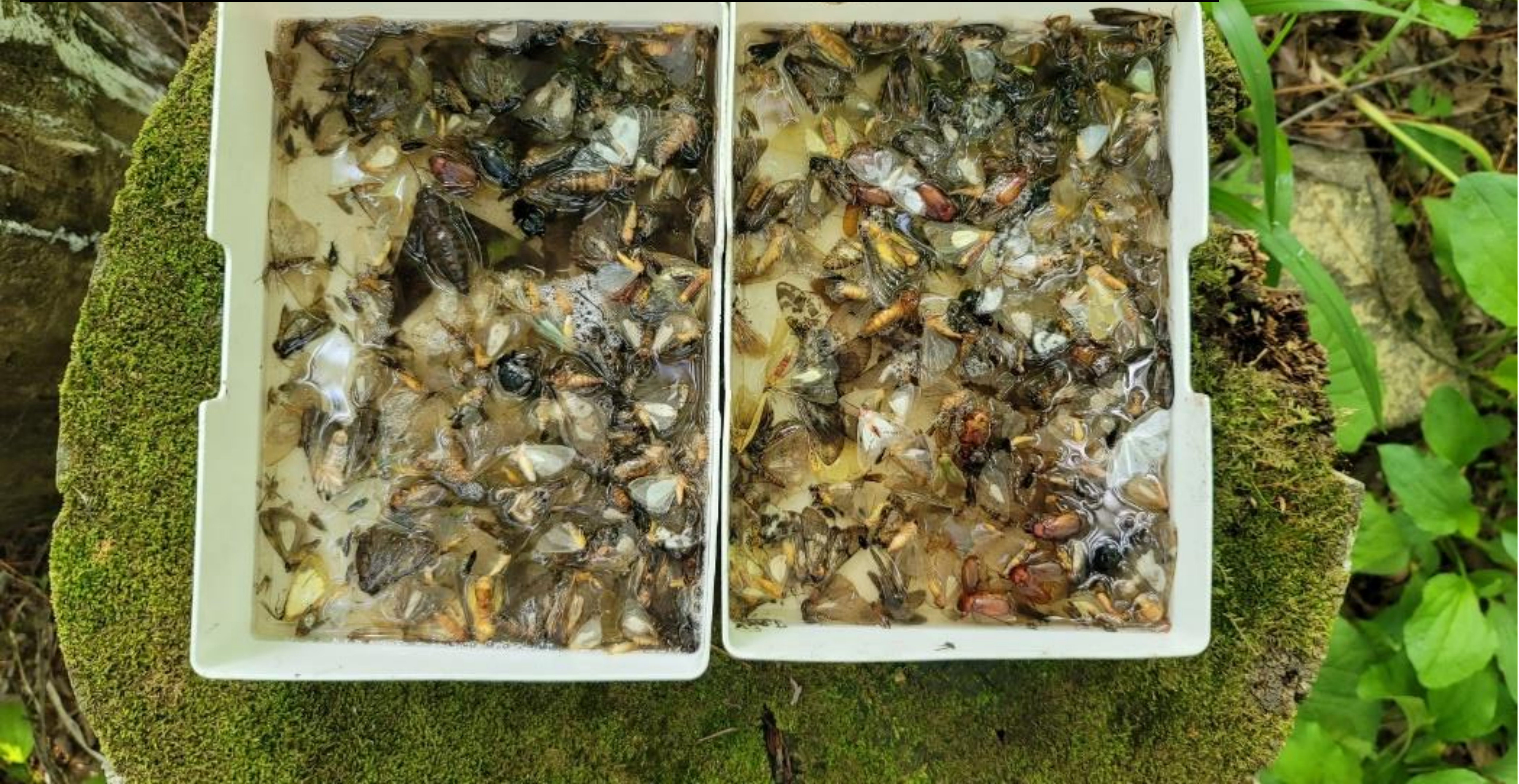

Figure S2. The sample condition of collected moths degraded due to fallen scales.
